# Social Determinants Predicting the Knowledge, Attitudes, and Practices of Women Toward Zika Virus Infection

**DOI:** 10.3389/fpubh.2020.00170

**Published:** 2020-06-03

**Authors:** Mari Kannan Maharajan, Kingston Rajiah, Jo-Ann Singco Belotindos, Marilou S. Basa

**Affiliations:** ^1^Department of Pharmacy Practice, School of Pharmacy, International Medical University, Kuala Lumpur, Malaysia; ^2^Master in Pharmacy Practice, School of Postgraduate Studies, International Medical University, Kuala Lumpur, Malaysia; ^3^College of Pharmacy, Southwestern University PHINMA, Cebu, Philippines

**Keywords:** zika virus, infection, social determinants, women, pregnancy, Philippines

## Abstract

**Objective:** To investigate the factors predicting knowledge, attitude, and practices (KAP) toward Zika virus infection among women population in Cebu City, Philippines.

**Study Design:** A cross-sectional survey was conducted from March 2018 to May 2018. Ethical practices were followed. A total of 702 women was approached and finally 516 completed the survey.

**Methods:** Descriptive analysis was undertaken for the participants' characteristics. Kolmogorov–Smirnov test was applied to declare the nature of data distribution. To determine the role of socio-demographic characteristics on KAP, differences in socio-demographic status were compared with the KAP scores using the one-way analysis of variance or Kruskal–Wallis test with *p* < 0.05 as significant. Logistic regression analysis was used to determine the predictors of each KAP domain (good and poor).

**Results:** There was a significant positive correlation between level of education and KAP scores. Also, there was a significant positive correlation between employment and KAP scores. Knowledge score was a significant predictor of practice score (*b* = 1.261, *p* = 0.024), and attitude score was also a significant predictor of practice score (*b* = 0.183, *p* = 0.039). However, knowledge score was not a significant predictor of attitude score (*b* = 0.316, *p* = 0.247).

**Conclusions:** The present findings provided an overall view of KAP on Zika virus infection among females in Philippines and the socio-demographic factors that affected their KAP. Women with postgraduate education and being in higher profession were the predictors influencing the KAP scores of this female population. Women with postgraduate education was the strongest predictor.

## Introduction

During a global outbreak, Zika virus infection spread rapidly across the world, and new cases of Zika virus infection were reported in Southeast Asia (1). It has become a major concern worldwide with a potential to cause a global pandemic during outbreak (2). Although Zika virus is transmitted through the bite of an infected mosquito of *Aedes* species, other modes of transmission such as maternal–fetal transmission or through sexual activity have received much attention (3). The severity of Zika virus infection has an impact on health, the economy, and social well-being, especially in pregnant women (4). One challenge of dealing with Zika virus infection in pregnant women is the lack of specific symptoms, as the infection can result in microcephaly in the fetus, which sometimes leads to stillbirths and miscarriages (5). In order to minimize Zika complications, in such pregnancy-related complications, it is important to diagnose at early stage (6). The World Health Organization (WHO) advocates the generation of evidence through research to strengthen guidance and action plans to minimize the spread of Zika virus infection and limit the impact of its complications (7).

Although Zika virus infection is not a public health emergency of international concern at this moment, if there is no active measures taken, transmission of this virus will continue to expand in the world, including Southeast Asia (8). Southeast Asian countries have a high prevalence of arboviral diseases, including Zika virus infection (9). The availability of habitat for *Aedes* mosquitoes and the ideal environmental conditions for the delivery of mosquito-borne pathogens have led the Southeast Asian region to face a huge health threat through mosquito-borne infectious diseases in the twenty-first century (10). The high prevalence of arboviral disease in this region has highlighted the need to combat the transmission of mosquito-borne pathogens (11).

In the Philippines, mosquito-borne diseases such as dengue, chikungunya, and Japanese encephalitis were reported earlier. During the global Zika outbreak, the Philippines experienced a notable number of cases (12). The rapid urbanization, industrialization-influenced lower sanitation, and favorable geographical location have led the distribution of mosquito-borne diseases (13). In addition, travelers from Zika virus-affected areas and from areas with abundant mosquitoes contributed to the transmission of Zika virus (14). The accumulated evidence on Zika virus infection and transmission among Filipinos has focused attention on the need for domestic awareness. Therefore, studies on health behavior or behavior change models including knowledge, attitudes and practice (KAP) may contribute to preventing the spread of infectious disease. No previous studies have examined the social determinants that predict the KAP of female populations in the Philippines toward Zika virus infection. An inferential study like this may assist healthcare professionals to develop appropriate precautionary measures to tackle Zika virus infection. Hence, the present study focused on variables such as age, relationship status, education, employment, monthly income, and type of household of the target population and its association with KAP.

## Methods

### Ethical Approval

The study was permitted by the Joint Committee for Ethics and Research of the study site [MPP 1/2018 (3)].

### Study Design

This cross-sectional study was conducted among women 18–45 years of age from March to May 2018 in Cebu City, Philippines. A minimum sample size of 374 was calculated using Raosoft software; power 80%, distribution of response 50%, with 95% confidence interval and a 5% margin of error. A total of 702 women was approached and finally 516 completed the survey; hence, the findings can be generalized. Participation in this survey was voluntary and informed consent was obtained in the front page of the questionnaire. Anonymity and confidentiality of participants were ensured. Simple random sampling was done to choose the areas in Cebu City. Convenience sampling was undertaken for house-to-house visits to recruit participants. A survey questionnaire was used to assess their KAP toward Zika virus infection. In each household, the questionnaire was administered to one family member who met the inclusion criteria; these criteria were Filipino women over the age of 18 living in Cebu City, Philippines, who had not participated previously in a similar survey.

### Study Questionnaire

The questionnaire, which was adapted from the WHO Resource pack (15), assessed KAP toward Zika virus infection. The questionnaire had 27 items divided into four sections: (i) demographic information, (ii) knowledge domain, (iii) attitudes domain, and (iv) practice domain toward Zika virus infection. The participants had to choose “Yes/No,” and a correct response was given a score of 1, whereas an incorrect response was given a score of 0. The participant's knowledge was calculated as the sum of correct responses and mean score was obtained. Higher scores indicated better knowledge. There were six items in the “attitudes” section. For each item, a five-point Likert scale was used (strongly agree, agree, neutral, disagree, and strongly disagree) in which strongly agree = 5 and strongly disagree = 1. The participants had to choose one of the options from the Likert scale. A high score was given when the statement defined better attitudes. The attitudes score was computed as the sum of participants' responses and mean score was obtained. There were five items in “practice” section. The participants had to choose “Yes/No”; for each correct response, a score of 1 was given, whereas an incorrect response was given a score of 0. The practice score was computed as the sum of participants' responses and mean score was obtained. Higher scores indicated better practice. The participants' KAP levels were defined as “good” or “poor” based on an 80% cutoff point (16, 17). For example, with a total score of 10 in the knowledge domain, a participant securing 8 or more was categorized as having good knowledge.

### Validity and Reliability of the Study Questionnaire

The questionnaire went through face and content validity by three subject experts and their inputs on the questionnaire were implemented. A pilot study was done on 20 samples to endorse the questionnaire reliability. The reliability was determined with reference to the Cronbach's alpha value by using SPSS V.23. In this study, Cronbach's alpha was 0.71 for knowledge, 0.72 for attitude, 0.79 for practice, and 0.75 for overall KAP. The pilot study data were not used for the final analysis.

### Data Analysis

Descriptive analysis was undertaken for the participants' characteristics. Kolmogorov–Smirnov test was applied to declare the nature of data distribution. To determine the role of socio-demographic characteristics on KAP, differences in socio-demographic status were compared with the KAP scores using the one-way analysis of variance or Kruskal–Wallis test with *p* < 0.05 as significant. Logistic regression analysis was used to determine the predictors of each KAP domain (good and poor). In the bivariate logistic regression, all independent variables (age, relationship status, education, employment, monthly income, and type of household) were included. In the next step, significant independent variables from the bivariate analysis (*p* ≤ 0.25) were entered into the multivariate analysis. Confounding factors were explored by comparing the difference between the adjusted odds ratio (aOR) in multivariate analyses and the crude odds ratio (OR) in bivariate analyses of each predictor variable on the KAP domain. The correlation values among KAP scores and between KAP scores and independent variables were calculated using Spearman's rank correlation keeping *p* < 0.01 as significant. Regression analysis was done to identify the strongest predictor variable among the socio-demographic characteristics. Data were analyzed using the SPSS version 24.

Before conducting regression, multicollinearity was checked to rule out the relationship among the independent variables. No significant correlations among independent variables were observed in the visual examination using scatter plots. The normality of the residual was verified by using the Kolmogorov–Smirnov test and the significant values were below 0.05, suggesting violations of the assumption of normality. As the data distribution was not normal, skewness of the data was analyzed. As the calculated *z*-value for skewness was less than the critical values of ±1.96 at 0.05 significance level, the distribution of data was considered normal (18). Linearity was checked using scatter plots. The scatter plot followed a linear pattern, which confirmed that linearity assumption was met. For equality of variance, the scatter plot that drew for linearity was used. Since the residuals did not appear in a triangular fashion, it was confirmed that the equal variance assumption was met.

## Results

### Study Population Characteristics

The socio-demographic characteristics of the study participants are represented in Table 1. Out of 516 respondents, 396 were under 35 years old. The majority (376) were in a relationship. A total of 398 participants were graduates, whereas 118 had undergone secondary schooling. More than half (375) were professionals and 141 were either laborers or jobless. The majority of respondents (333) reported having an average monthly income of USD 300 (approximately Philippine peso 15,666.21) or less. More than 50% of the respondents (272) were living in apartments or high-grade housing, whereas the rest were living in low-cost housing or lower-grade households.

**Table 1:** Socio-demographic characteristics of the participants.

**Characteristics of respondents**	***n* (%)**	**Mean score**		
		**Knowledge (*SD*)**	**Attitude (*SD*)**	**Practice (*SD*)**
**Age (in years)**				
18–24	181 (35.1)	6.6 (2.34)	22.2 (4.34)	3.2 (3.45)
25–30	133 (25.8)	7.1 (1.98)	23.5 (4.73)	3.3 (3.97)
31–35	82 (15.9)	7.2 (2.51)	23.6 (5.48)	3.1 (4.02)
>35	120 (23.3)	7.2 (1.87)	23.8(4.97)	3.3 (3.93)
**Relationship status**				
Single and not in any relationship	140 (27.1)	5.8 (3.15)	21.3 (3.98)	2.8 (2.91)
Married	223 (43.2)	6.2 (2.79)	22.8 (4.08)	3.1 (3.03)
Living together	94 (18.2)	7.1 (2.46)	23.1 (4.23)	2.9 (3.14)
Married but living separately	12 (2.3)	7.3 (1.84)	23.4(5.02)	3.2 (2.96)
Divorced	47 (9.1)	6.8 (2.42)	22.7 (4.72)	2.7 (3.01)
**Level of education**				
Elementary	102 (29.8)	4.5 (1.49)	20.6 (3.89)	2.4 (3.18)
Secondary	16 (3.1)	5.2 (2.21)	21.3 (4.57)	2.6 (3.25)
Graduate	370 (71.7)	6.9 (2.02)	23.6 (4.84)	3.1 (2.71)
Postgraduate	28 (5.4)	8.2 (1.82)	25.3 (4.21)	4.2 (2.83)
**Employment**				
Jobless	68 (13.2)	4.2 (1.73)	20.3 (3.56)	2.4 (3.02)
Unskilled labor	39 (7.6)	5.1 (2.34)	21.5 (4.21)	2.6 (2.95)
Skilled labor	34 (6.6)	5.8 (3.12)	21.8 (3.87)	2.7 (3.16)
Professional	114 (22.1)	6.7 (2.84)	22.5 (3.61)	3.1 (2.89)
Manager	261 (50.6)	8.1 (1.68)	25.1 (4.83)	4.2 (3.09)
**Monthly income (USD)**				
<200	185 (35.5)	4.6 (3.16)	20.1 (3.45)	2.5 (2.78)
201–300	148 (28.7)	4.9 (2.49)	20.9 (3.89)	2.5 (2.83)
301–400	45 (8.7)	5.5 (2.87)	21.4 (3.24)	2.6 (3.21)
401–500	67 (13.0)	6.2 (2.54)	21.7 (3.82)	2.7 (2.80)
501–600	59 (11.4)	6.8 (2.82)	22.2 (4.57)	2.9 (2.94)
>600	12 (2.3)	7.1 (2.43)	22.8 (3.12)	3.1 (3.18)
**Type of household**				
Apartment	113 (21.9)	6.1 (3.62)	21.3 (4.65)	2.8 (3.04)
Low-cost housing	154 (29.8)	4.8 (2.74)	21.2 (4.81)	2.6 (2.86)
Informal housing	53 (10.3)	4.9 (2.93)	20.6 (4.28)	2.5 (3.09)
Condominium	10 (1.9)	5.5 (2.86)	22.4 (3.91)	3.0 (2.88)
Bungalow	121 (23.4)	6.2 (2.47)	22.8 (4.02)	3.1 (2.90)
Lodging house	50 (9.7)	5.6 (2.32)	21.9 (3.18)	2.7 (3.17)
Others	15 (2.9)	5.2 (3.32)	22.0 (3.73)	2.9 (3.05)

### Knowledge on Zika Virus Infection, Spread, and Management

A statistically significant difference in the mean knowledge scores was identified between levels of education (*p* < 0.001). The highest mean knowledge score was obtained by the women who had postgraduate education qualification and the lowest mean knowledge score was obtained by the women who had elementary level education. Also, a statistically significant difference in the mean knowledge score was identified between levels of employment (*p* < 0.001). The highest mean knowledge score was obtained by the women in managerial position and the lowest by the jobless women (Table 1). Each independent variable was first entered separately into bivariate logistic regression models to evaluate the association with socio-demographic characteristics. Variables such as age group of 31–35 years, living together relationship, postgraduate level of education, employed as manager, and high monthly income had significant associations (*p* < 0.05). Type of household had no association with participants' knowledge (Table 2). All the variables were then entered in multivariate logistic regression. After excluding insignificant predictor variables (*p* > 0.25) from the analysis, the multivariate model revealed that the level of education and employment status were the two independent predictor variables of knowledge (Table 2). The results revealed that there was an increased odds of having good knowledge among participants who have postgraduate education, compared to the elementary education with OR (95% CI): 2.51 (2.06–2.48), and having good knowledge among participants who were manager, compared to the jobless with OR (95% CI): 3.01 (1.82–2.19).

**Table 2:** Odds ratios of knowledge scores with socio-demographic characteristics (good vs. poor).

**Independent variable**	**Bivariate**		**Multivariate**	
	**Crude odds ratio (95% CI)**	***p*-value**	**Adjusted odds ratio (95% CI)**	***p*-value**
**Age (in years)**		0.028		0.438
18–24	1		1	
25–30	2.28 (1.43–1.87)		1.96 (0.32–0.56)	0.035*
31–35	2.15 (1.52–1.76)		1.99 (0.55–1.28)	0.042*
>35	1.31 (1.32–1.93)		1.21 (1.02–1.87)	0.158
**Relationship status**		0.041		0.058
Single and not in any relationship	1		1	
Married	1.15 (0.64–1.23)		1.04 (0.42–1.65)	0.296
Living together	1.21 (1.02–2.14)		1.32 (1.34–2.23)	0.021*
Married but living separately	1.02 (1.43–2.21)		1.29 (1.28–1.41)	0.483
Divorced	1.10 (0.43–0.97)		1.02 (1.23–1.74)	0.369
**Level of education**		<0.001		0.003*
Elementary	1		1	
Secondary	1.14 (1.23–2.56)		1.05 (0.93–1.62)	0.360
Graduate	1.53 (0.87–1.43)		2.02 (0.71–1.13)	0.052
Postgraduate	2.02 (1.76–2.14)		2.51 (2.06–2.48)	0.013*
**Employment**		<0.001		0.002*
Jobless	1		1	
Unskilled labor	1.02 (1.32–2.14)		1.10 (1.25–1.48)	0.347
Skilled labor	1.25 (1.56–2.32)		1.11 (0.62–1.29)	0.512
Professional	1.93 (0.76–1.57)		2.03 (1.28–1.70)	0.494
Manager	3.42 (1.82–2.19)		3.01 (1.82–2.19)	0.012*
**Monthly income (USD)**		0.030		0.428
<200	1		1	
201–300	1.29 (1.23–1.52)		0.99 (0.43–1.32)	0.384
301–400	1.33 (1.24–1.98)		1.03 (0.54–0.98)	0.512
401–500	1.54 (2.13–3.82)		1.04 (1.43–1.85)	0.419
501–600	1.56 (1.62–1.93)		1.46 (0.67–1.23)	0.376
>600	1.79 (0.81–1.36)		1.78 (1.98–2.43)	0.037*
**Type of household**		0.604	**-**	**-**
Apartment	1			
Low-cost housing	0.73 (1.46–1.98)			
Informal housing	0.68 (0.87–1.53)			
Condominium	1.49 (0.98–1.74)			
Bungalow	1.52 (1.72–2.48)			
Lodging house	0.84 (1.98–2.64)			
Others	1.02 (0.43–0.87)			

* *p < 0.05*.

### Attitudes Toward Zika Virus Infection

A significant difference in the mean attitude scores was identified between levels of education (*p* = 0.04). The highest mean attitude score was obtained by the women who had postgraduate degree and the lowest was obtained by the women who had elementary level education (Table 1). Each independent variable was first entered separately into bivariate logistic regression models to evaluate the association with socio-demographic characteristics. Variables such as postgraduate level of education, employed as manager, and high monthly income had significant associations (*p* < 0.05). Other variables had no association with participants' attitude (Table 3). All the variables were then entered in multivariate logistic regression. According to this, none of the variables was a predictor of participants' attitudes toward Zika virus infection (Table 3).

**Table 3:** Odds ratios of attitude scores with socio-demographic characteristics (good vs. poor).

**Independent variable**	**Bivariate**		**Multivariate**	
	**Crude odds ratio (95% CI)**	***p*-value**	**Adjusted odds ratio (95% CI)**	***p*-value**
**Age (in years)**		0.387	**–**	**–**
18–24	1			
25–30	0.89 (1.27–1.64)			
31–35	0.97 (0.85–1.21)			
>35	1.18 (0.29–0.72)			
**Relationship status**		0.635	**–**	**–**
Single and not in any relationship	1			
Married	0.76 (1.42–1.63)	0.371		
Living together	0.71 (0.28–0.84)	0.026		
Married but living separately	1.23 (0.37–1.12)	0.261		
Divorced	1.09 (0.35–0.78)	0.482		
**Level of education**		0.037		0.423
Elementary	1		1	
Secondary	1.02 (0.58–0.93)		1.10 (0.67–1.23)	0.295
Graduate	1.26 (1.23–1.56)		1.19 (1.13–1.84)	0.052
Postgraduate	1.34 (0.76–1.42)		1.32 (1.02–1.43)	0.045*
**Employment**		0.014		0.245
Jobless	1		1	
Unskilled labor	1.13 (0.62–1.43)		1.10 (1.25–1.48)	0.368
Skilled labor	1.20 (1.23–1.56)		1.11 (0.62–1.29)	0.492
Professional	1.85 (1.24–1.68)		1.13 (0.86–1.17)	0.327
Manager	2.12 (1.82–2.19)		1.21 (1.52–2.02)	0.041*
**Monthly income (USD)**		0.039		0.356
<200	1		1	
201–300	0.99 (0.43–1.32)		0.98 (0.83–1.21)	0.347
301–400	1.03 (0.54–0.98)		0.99 (0.46–1.37)	0.285
401–500	1.04 (1.43–1.85)		0.98 (0.69–0.92)	0.416
501–600	1.46 (0.67–1.23)		0.99 (0.23–0.49)	0.371
>600	1.78 (1.98–2.43)		1.02 (1.89–2.13)	0.065
**Type of household**		0.373	**–**	**–**
Apartment	1			
Low-cost housing	0.62 (0.46–1.23)			
Informal housing	0.64 (0.72–1.37)			
Condominium	1.26 (1.42–1.69)			
Bungalow	1.37 (0.62–1.28)			
Lodging house	0.76 (0.87–1.48)			
Others	0.92 (0.84–1.58)			

* *p < 0.05*.

### Zika Virus Infection Prevention Practices

A statistically significant difference in the mean practice scores was identified between levels of education (*p* = 0.028). The highest mean practice score was obtained by the women who had postgraduate education and the lowest was obtained by the women who had elementary level education. Also, a statistically significant difference in the mean practice score was identified between levels of employment (*p* = 0.021). The highest mean practice score was obtained by the women in managerial position and the lowest was obtained by the jobless women (Table 1). Each independent variable was first entered separately into bivariate logistic regression models to evaluate the association with socio-demographic characteristics. Variables such as postgraduate level of education, employed as manager, and high monthly income had significant associations (*p* < 0.05). Other variables had no association with participants' preventive practice (Table 4). All the variables were then entered in multivariate logistic regression to get adjusted odds ratios, which will reveal the predictors. After excluding insignificant predictor variables (*p* > 0.25) from the analysis, the multivariate model revealed that level of education and employment status were the independent predictor variables of practice (Table 4). The results revealed that there was increased odds of having good practice among participants who have postgraduate education, compared to the elementary education with OR (95% CI): 2.64 (1.02–1.43), and increased odds of having good practice among participants who were manager, compared to the jobless with OR (95% CI): 3.02 (1.61–2.52).

**Table 4:** Odds ratios of practice scores with socio-demographic characteristics (good vs. poor).

**Independent variable**	**Bivariate**		**Multivariate**	
	**Crude odds ratio (95% CI)**	***p*-value**	**Adjusted odds ratio (95% CI)**	***p*-value**
**Age (in years)**		0.421	**-**	**-**
18–24	1			
25–30	1.10 (0.67–1.42)			
31–35	1.17 (0.45–0.81)			
>35	1.23 (1.25–1.71)			
**Relationship status**		0.413	**-**	**-**
Single and not in any relationship	1			
Married	1.01 (0.53–1.08)			
Living together	1.11 (1.14–1.48)			
Married but living separately	1.13 (0.47–0.72)			
Divorced	1.04 (1.37–1.56)			
**Level of education**		<0.001		0.004*
Elementary	1		1	
Secondary	1.21 (1.31–1.34)		1.11 (1.67–2.13)	0.349
Graduate	1.94 (0.63–1.26)		2.05 (1.35–1.91)	0.058
Postgraduate	2.73 (0.67–1.23)		2.64 (1.02–1.43)	0.027*
**Employment**		<0.001		0.002*
Jobless	1		1	
Unskilled labor	1.51 (0.73–1.30)		1.04 (1.54–1.87)	0.259
Skilled labor	1.55 (0.83–1.29)		1.16 (1.29–1.83)	0.285
Professional	2.59 (2.24–2.62)		2.44 (0.61–0.76)	0.063
Manager	3.24 (0.76–1.13)		3.02 (1.61–2.52)	0.021*
**Monthly income (USD)**		0.045	**–**	0.358
<200	1		1	
201–300	1.02 (0.34–0.82)		1.04 (1.26–1.74)	0.468
301–400	1.57 (1.54–1.98)		1.08 (0.67–1.01)	0.352
401–500	1.82 (0.43–0.85)		1.56 (1.32–1.23)	0.195
501–600	2.66 (1.67–1.93)		2.02 (0.98–1.36)	0.073
>600	2.98 (0.98–1.43)		2.98 (0.71–1.34)	0.041*
**Type of household**		0.681	**-**	**-**
Apartment	1			
Low-cost housing	1.02 (1.46–1.83)			
Informal housing	0.98 (1.72–2.17)			
Condominium	1.45 (0.42–1.09)			
Bungalow	1.75 (1.23–1.58)			
Lodging house	0.99 (1.72–2.34)			
Others	0.95 (1.43–1.86)			

* *p < 0.05*.

### Correlation Among KAP Scores, Between KAP Scores and Independent Variables

After identifying the predictor variables (level of education, employment), correlation analysis was done among KAP scores, between KAP scores and predictor variables. There was a significant positive correlation between level of education and knowledge and practice scores but not attitude scores. Also, there was a significant positive correlation between employment and knowledge and practice scores but not attitude scores (Table 5). The significant correlations between knowledge and practice scores with level of education indicated that knowledge and practice regarding Zika virus infection increased with increasing education level. Likewise, the significant correlations between knowledge and practice scores with employment indicated that knowledge and practice regarding Zika virus infection increased with increasing level of employment.

**Table 5:** Correlation among participants' scores on knowledge, attitude, and practice and between knowledge, attitudes, and practice; level of education; and employment.

**Variables**	**Correlation coefficient**	***p*-value**
Knowledge-attitudes	0.32	<0.001*
Knowledge-practice	0.36	0.003*
Attitudes-practice	0.24	0.004*
Level of education-knowledge	0.45	<0.001*
Level of education-attitudes	0.14	0.315
Level of education-practice	0.29	0.003*
Employment-knowledge	0.42	<0.001*
Employment-attitudes	0.15	0.402
Employment-practice	0.28	0.003*

* *Correlation significant at the 0.01 level (two-tailed)*.

There was also a significant positive correlation between knowledge–attitude, knowledge–practice, and attitude–practice with the strongest correlation identified for knowledge–practice. To validate these results, regression analysis was conducted using knowledge, attitude, and practice scores.

### Regression Analysis of Knowledge and Attitude Scores Against Practice Score

First, regression analysis was performed using attitudes score as the outcome variable and the variables knowledge score, practice score as predictors (Supplementary File 1). Practice score was a significant predictor of attitudes score (*b* = 0.169, *p* = 0.045). However, knowledge score was not a significant predictor of attitudes score (*b* = 0.316, *p* = 0.247). Further regression analysis was done by keeping knowledge and attitudes scores in the model (Table 6). By controlling attitudes score, it was found that practice score increased by 1.26 for every unit increase in knowledge score (*b* = 1.261, *p* = 0.024). By controlling knowledge score, practice score increased by 0.18 for every increased unit in attitude score (*b* = 0.183, *p* = 0.039). The regression results revealed that knowledge of participants does not contribute to the attitudes of participants though attitude contributes to practice to some extent. However, knowledge on Zika virus infection plays a key role in improving the prevention practices toward Zika virus infection. Hence, knowledge score was regressed stepwise against the two predictor variables (level of education and employment) to analyze any significant change occurring in knowledge score.

**Table 6:** Multivariate regression of knowledge and attitude scores against practice score.

**Variables**	**Unstandardized coefficient**		**Standardized coefficient**		
	**B weight (*b*)**	**Standard error**	**Beta**	***t***	***p*-value**
Knowledge	1.261	1.158	0.874	6.473	0.024*
Attitude	0.183	1.281	0.726	4.680	0.039*

* *p < 0.05*.

### Stepwise Regression for Knowledge Score

Stepwise regression is a method of fitting regression models in which the choice of predictive variables is carried out by an automatic procedure. In each step, an independent variable is considered for addition to or subtraction from the set of independent variables. Each model is autogenerated by the SPSS during regression. The non-significant contributors were excluded automatically by the SPSS. Mahalanobis distance is used to investigate outlier and the value 6.34 is within the limit.

Table 7 shows the stepwise regression for knowledge score. Only two models significantly affected the knowledge scores. In model 1, elementary education of the participants alone accounted for 31.3% of the variance in knowledge scores. In model 2, postgraduate education and manager position of the participants together contributed 43.8% of the variance in knowledge scores. Model 2 was selected in this study, as the adjusted *R*^2^-value was higher (43.8% explained variance) than model 1. In model 2, there was a significant *R*^2^ change of 12.5% variance. To determine the strongest predictor, individual coefficients were analyzed. The standardized coefficient beta value of postgraduate education was 0.423 and confirmed that it was the best predictor. The shared and unique contribution of the predictors have been analyzed. “Postgraduate education” has shared (0.458)^2^ = 20.9%, unique (0.376)^2^ = 14.1% to the knowledge score. “Manager position” has shared (0.412)^2^ = 16.9%, unique (0.352)^2^ = 12.3% contribution to the knowledge score (Table 8).

**Table 7:** Stepwise regression for knowledge score.

**Regression model**	***R*^**2**^**	***R*^**2**^ change**	**Adjusted *R*^**2**^**	**Standard error of the estimate**	***F* change**
Model 1^a^	0.332	0.332	0.313	0.469	4.781
Model 2^b^	0.457	0.125	0.438	0.314	3.652

a *Postgraduate education*.

b *Postgraduate education, manager position*.

**Table 8:** Coefficient table for unique and shared contributions of predictors.

**Model**		**Unstandardized coefficient**		**Standardized coefficient**			**Correlations**			**Collinearity statistics**	
		**B weight**	**Standard error**	**Beta**	***t***	***p*-value**	**Zero order**	**Partial**	**Part**	**Tolerance**	**VIF**
2	Postgraduate education	0.423	1.278	0.374	3.681	<0.001*	0.558	0.458	0.376	0.264	1.271
	Manager position	0.307	1.025	0.301	3.194	<0.001*	0.483	0.412	0.352	0.196	1.148

* *p < 0.05*.

## Discussion

The clinical consequences of Zika virus infection and the well-connected global transportation system that allows for the possible global spread of Zika virus are the major challenges (19). The tropical and sub-tropical climate of Southeast Asia and its heterogeneity in socio-economic factors make it vulnerable to mosquito-borne infectious diseases (20, 21). Considering these facts, many types of interventions have been implemented to deal with people with suspected and actual Zika virus infection (22). It is essential to measure the effectiveness of such interventions. The result of the present study, the first one from the Philippines, assessed the knowledge among reproductive-age women in the Philippines about Zika virus infection and evaluated the factors contributing to their attitude and preventive practices in dealing with transmission of the virus. The predominant dispersion of the Aedes genus in tropical areas emphasizes the importance of developing an efficient strategy to prevent the spread of the vectors (23). Knowledge of the general public and their understanding of the consequences of Zika virus infection will help to reduce the health consequences of Zika virus infection (24) and to increase in populations' willingness to pay for a future vaccination (25). The 73.5% response rate suggested that there was no participation bias and was an indication of the willingness of the study population to participate in research related to Zika virus infection.

Factors associated with good knowledge regarding Zika virus infection were age group, relationship status, level of education, monthly income, and employment. In terms of age group, women within the age of 25–35 years were almost two times more likely to have good knowledge compared to women within the age of 18–24 years. This suggested that the women between 25 and 35 years are in reproductive age and they are aware of Zika virus infection, as it affects them and can cause fetal injury and pregnancy complications. Considering the complications associated with Zika virus infection during pregnancy, knowledge of women in this age group is an important element to prevent microcephaly and other birth defects. In terms of the relationship status, women who were in living together relationship were 1.3 times more likely to have good knowledge compared to women who are single and not in any relationship. This suggested that those who are in a relationship would have updated themselves about Zika virus infection. This agrees with findings from studies in other Zika virus-affected countries (26, 27). The level of knowledge in women who are in a relationship should intuitively be high, as they are likely to have babies at some stage of their lives in the near future. Overall, the study participants who were in a relationship and in childbearing age were aware of the risk of Zika virus infection. These results demonstrate that females in the Philippines with potential exposure to Zika virus have awareness of Zika virus infection. In terms of monthly income, women who were earning more than 600 USD per month were 1.8 times more likely to have good knowledge compared to women who were earning <200 USD per month. This suggested that women who are at low socio-economic status have poor awareness toward Zika virus infection. Socio-economic conditions are major factors involved in the spread of infectious diseases, especially those that are vector-borne (28). A global brief on vector-borne diseases mentioned that developing countries and regions with a low socio-economic status are susceptible (29). However, our multivariate regression analysis revealed that participants' level of education and employment were the predictors for the knowledge domain regarding Zika virus infection. In terms of level of education, women with postgraduate education were 2.5 times more likely to have good knowledge compared to women with elementary education. This finding that women who had less education were significantly less knowledgeable about Zika virus infection is similar to that of Guo et al. (30). However, Abramson and Piltch-Loeb reported that there was no association of knowledge related to Zika virus infection of women of childbearing age and their level of education with Zika knowledge (31). Wilhelmova et al. reported that health literacy and a healthier lifestyle are associated with a higher level of education (32). In order to prevent Zika virus infection and its related complications, there is a need to educate everyone irrespective of their educational background, age, and sex. The limited data available to guide health awareness strategy, along with how to improve health literacy is one concern in the efforts to prevent Zika virus infection. Therefore, special attention should be paid to the people with lower educational background and a strategy should be developed to create awareness about Zika virus infection. In terms of employment, women who were in manager position were three times more likely to have good knowledge compared to women who were jobless. Awareness and knowledge about Zika virus infection in women who have jobs can increase over time through public health education and messaging. The women who are educated and employed might have received more opportunities for seeking and obtaining information about Zika virus infection with targeted campaigns through healthcare providers and through online resources and digital outreach. Their accessibility to internet and healthcare providers has served as effective avenues for disseminating knowledge about Zika virus infection (33).

Factors associated with good attitudes toward Zika virus infection were level of education employment and monthly income. In terms of level of education, women with postgraduate education were 1.3 times more likely to have good attitudes compared to women with elementary education. In terms of employment, women in manager position were 1.2 times more likely to have good attitudes compared to women who were jobless. In terms of monthly income, there was no significant variation in attitudes among women with respect to their monthly earning. However, our multivariate regression analysis revealed that none of the factors was a predictor for the attitudes domain regarding Zika virus infection. This result indicated that none of the participants' socio-demographic characteristics affected their attitudes toward Zika virus infection.

Factors associated with good practices toward Zika virus infection were level of education, employment, and monthly income. In terms of monthly income, women who were earning more than 600 USD per month were almost three times more likely to have good practice compared to women who were earning <200 USD per month. This suggested that women who are in low socio-economic have poor prevention practices toward Zika virus infection. However, our multivariate regression analysis revealed that participants' level of education and employment were the predictors for the practice domain regarding Zika virus infection. In terms of level of education, women with postgraduate education were 2.6 times more likely to have good practice compared to women with elementary education. In terms of employment, women who were in manager position were three times more likely to have good practice compared to women who were jobless.

This study revealed that knowledge and practices had correlations and it was found that prevention practices toward Zika virus infection increased drastically with increase in knowledge of Zika virus infection. However, though attitudes and practice had correlations, there was very minimal increase in practices toward Zika virus infection. In addition to this, the results suggested that knowledge was not predicting the attitudes. This finding revealed that better knowledge does not necessarily give better attitudes. A similar result was reported by the study of Alobuia et al. (34). The present study also revealed education as the best predictor for knowledge toward Zika virus infection. This emphasizes the need for the government and the Ministry of Health officials to find ways of providing and improving access to health promotion and disease prevention educational materials. Such programs across the country aim to provide opportunity for citizens to understand the risks of vector-borne diseases and promote prevention practices to prevent transmission of Zika virus infection. The data from this study will be valuable in the planning of tailored health education for the prevention of Zika virus infection. The study results also highlight the awareness of people about fetal safety, especially those who are of childbearing age.

## Limitations

A self-administered questionnaire was used; this may have influenced the results because of the inevitable social desirability bias. The cross-sectional study design may result in a lack of causal inference. As this study instrument is a written form, it did not provide an opportunity to those who may be unable to read. The questionnaire did not ask the participants about their or their partners' recent international travel to Zika virus-affected areas, as these may influence their KAP toward Zika virus infection.

## Conclusions

The present findings provided an overall view of KAP on Zika virus infection among females in the Philippines and the socio-demographic factors that affected their KAP. Women with postgraduate education and being in a higher profession were the predictors influencing the knowledge scores of this female population, which is linked to their prevention practices toward Zika virus infection. Women with postgraduate education was the strongest predictor. On the other hand, women who are at low socio-economic status have poor awareness toward Zika virus infection. There is a need for educating the women population through various campaigns and other public health measures to create greater awareness and better practices toward Zika virus infection prevention in the Philippines. The findings from this study have important public health implications for health promotion in the Philippines.

## Data Availability Statement

The datasets generated for this study are available on request to the corresponding author.

## Ethics Statement

The studies involving human participants were reviewed and approved by International Medical University—Joint Committee on Research and Ethics [MPP 1/2018 (3)]. The patients/participants provided their written informed consent to participate in this study.

## Author Contributions

KR, MM, and J-AB conceived and designed the study. MB and J-AB conducted research, provided research materials, and collected and organized data. KR and MM analyzed and interpreted the data and revised the manuscript. J-AB wrote an initial and final draft of the manuscript, and provided logistic support. All authors have critically reviewed and approved the final draft, and are responsible for the content and similarity index of the manuscript.

## Conflict of Interest

The authors declare that the research was conducted in the absence of any commercial or financial relationships that could be construed as a potential conflict of interest.
