# Supplementary material for: Social Determinants Predicting the Knowledge, Attitudes, and Practices of Women Toward Zika Virus Infection
Source: Front Public Health. 2020 Jun 3;8:170. doi: 10.3389/fpubh.2020.00170 (PMC7286053; doi:10.3389/fpubh.2020.00170)
Supplement: Supplementary file 1 [file Table_1.DOCX]

**Supp. File 1: Multivariate regression of knowledge and practice scores against attitudes score**

| **Variables** | **Unstandardized coefficient** | | **Standardized coefficient** |  |  |
| --- | --- | --- | --- | --- | --- |
|  | **B weight (b)** | **Standard error** | **Beta** | **t** | **Sig.** |
| Knowledge | .316 | .082 | .054 | 1.364 | .247 |
| Practice | .169 | 1.051 | .283 | 1.036 | .045 |

*p<0.05
